# Task-domain and hemisphere-asymmetry effects in cisgender and transmale individuals

**DOI:** 10.1371/journal.pone.0260542

**Published:** 2021-12-07

**Authors:** E. Darcy Burgund

**Affiliations:** Department of Psychology, Macalester College, Saint Paul, Minnesota, United States of America; University of Lausanne, SWITZERLAND

## Abstract

The present research examined the extent to which transmale individuals’ functional brain organization resembles that of their assigned sex or gender identity. Cisgender-female, cisgender-male, and transmale participants, who were assigned female sex but did not have a female gender identity, were compared in terms of effects that have been observed in cisgender individuals: task-domain effects, in which males perform better than females on spatial tasks and females perform better than males on verbal tasks; and hemisphere-asymmetry effects, in which males show larger differences between the left and right hemispheres than females. In addition, the present research measured participants’ intelligence in order to control for potential moderating effects. Participants performed spatial (mental rotation) and verbal (lexical decision) tasks presented to each hemisphere using a divided-visual field paradigm, and then completed an intelligence assessment. In the mental-rotation task, cismale and transmale participants performed better than cisfemale participants, however this group difference was explained by intelligence scores, with higher scores predicting better performance. In the lexical-decision task, cismale and transmale participants exhibited a greater left-hemisphere advantage than cisfemales, and this difference was not affected by intelligence scores. Taken together, results do not support task-domain effects when intelligence is accounted for; however, they do demonstrate a hemisphere-asymmetry effect in the verbal domain that is moderated by gender identity and not assigned sex.

## Introduction

The 21^st^ century has brought increased awareness of people whose gender identity is different from that associated with the sex they were assigned at birth. Sex is assigned at birth based on the appearance of genitalia and is almost always “female” or “male” [1]. People who do not identify as their assigned sex may identify as the opposite gender (e.g., assigned female sex at birth and identify as male gender) or may not identify with either binary female/male gender category [2]. The term “transgender” may be used to describe all people whose gender identity is different from the sex they were assigned at birth. In contrast, the term “cisgender” is used to describe people whose gender identity is the same as their assigned sex.

Transgender identities are hypothesized to emerge due to atypical sex hormones during critical pre- and postnatal developmental periods that cause sexual differentiation of the brain to deviate from genitalia development [3]. Certain aspects of cognitive function are shaped during these periods of brain development, and lead to differences between cisgender females and males that can be observed in adulthood within particular cognitive domains [4, 5]. As such, the extent to which functional neuroanatomy in transgender individuals is organized in alignment with expressed gender identity or assigned sex may be evaluated, in part, by examining performance in cognitive domains that exhibit differences between cisgender females and males.

Although cisgender females and males are highly similar in most areas [6], two potential differences have received considerable attention [7]. One is a difference in task domain (referred to as the “cognitive criterion” by Hirnstein et al. [2019] [7]), in which males perform better than females on spatial tasks, especially those involving mental rotation, and females perform better than males on verbal tasks. The other is a difference in hemisphere asymmetry (the “hemispheric asymmetry criterion” [7]), in which cisgender males exhibit a greater difference between the right and left hemispheres than cisgender females in tasks that show hemisphere biases. In particular, males exhibit a greater right-hemisphere advantage than females during spatial tasks and a greater left-hemisphere advantage than females during verbal tasks. Support for task-domain and hemisphere-asymmetry effects in cisgender individuals is not entirely consistent. In terms of task-domain effects, many studies have observed greater spatial performance in males than females [8–10] or greater verbal performance in females than males [11–13]; nonetheless, these differences are affected significantly by related factors, such as activities engaged in as a child [14] and implicit stereotypes regarding gender and ability [15]. In terms of hemisphere-asymmetry effects, some studies have observed greater asymmetries in males than females in spatial [16–19] or verbal [20–22] tasks, however other research observes small [23], contradictory [24], or nonexistent differences [25–28].

Despite the lack of consistency in research with cisgender individuals, task-domain and hemisphere-asymmetry effects have been used to evaluate the extent to which transgender individuals’ functional neuroanatomy is moderated by assigned sex or gender identity. Some studies suggest that transgender individuals’ performance is more like that of their assigned sex than that of their gender identity. In terms of task-domain effects, Cohen-Kettenis et al. (1998) [29] and Haraldsen et al. (2005, 2003) [30, 31] observed greater performance on spatial tasks in participants assigned male at birth than participants assigned female at birth, regardless of their gender identity. In terms of hemisphere asymmetries, Wisniewski et al. (2005) [32] observed a similar left-hemisphere advantage for letter identification in people assigned male at birth regardless of their male (cisgender) or female (transgender) gender identity. Other studies, in contrast, suggest that transgender individuals’ performance is more like that of their gender identity than that of their assigned sex. In terms of task-domain effects, transgender females performed better on verbal tasks than transgender males [29, 33], and transgender males performed better on spatial tasks than transgender females [34]. In terms of hemisphere asymmetries, cisgender males exhibited a greater left hemisphere advantage in a dichotic listening task than cisgender and transgender females, which did not differ [35]. Thus, while task-domain and hemisphere-asymmetry differences have been compared in cisgender and transgender participants, the extent to which these effects align more with assigned sex or gender identity is unclear.

There are many factors that may contribute to the inconsistencies in previous research. One factor is whether or not transgender participants are undergoing gender-affirming hormone therapy (i.e., taking estrogen or testosterone to affirm a more feminine or masculine identity, respectively). It seems probable that transgender individuals undergoing hormone therapy would be more likely to exhibit effects that align with their gender identity than individuals who are not undergoing therapy. Critically, however, this does not appear to be the case, as some studies in which participants are not taking hormones observed effects that align more with gender identity [29, 33], and other studies in which participants are taking hormones observed effects that align more with sex [30, 32]. Moreover, studies directly comparing the same participants before and after beginning hormone therapy have not observed changes in performance [30, 31, 36]. Thus, while the presence or absence of hormone therapy is an important factor to consider, it cannot be the only explanation for the inconsistencies in previous findings.

Other factors that may contribute to the inconsistencies in previous research concern limitations to the studies themselves. For one, some studies contain fewer than 15 participants in one or more of their participant groups [30, 31, 33, 34, 36], and as such, may not have adequate statistical power to detect important differences. Also problematic, many studies include wide age ranges within participant groups. For example, Cohen-Kettenis et al. (1998) [29] included participants who were 18–57 years old; Govier et al. (2010) [35] included participants who were 19–72 years old; and others have included ranges with standard deviations of 9 years and greater [30, 31, 36]. Spatial abilities [37, 38], verbal abilities [39, 40], and hemisphere asymmetries [41, 42] are known to change with age; thus, averaging across wide age ranges has the potential to blur relevant effects. Finally, some studies confound cis- and transgender group with age and education level, e.g., transgender participants are older and more educated than cisgender participants [30, 31] or cisgender participants are older and more educated than transgender participants [32]. As stated in recent reviews [4, 43], these limitations must be overcome for a clear picture of (trans)gender functional neuroanatomy.

Also crucial for the understanding of gender functional neuroanatomy, Hines (2020) [4] argues, is the measurement and control of participants’ intelligence, which has been largely neglected by research on transgender cognition and functional organization. As described by Hines (2020) [4], intelligence is important to include in studies of gender because female gender and higher intelligence relate positively to whether someone volunteers to participate in research, leading male volunteers to be more intelligent and educated than female volunteers. Selection biases related to intelligence may be especially pronounced in studies comparing transgender and cisgender participants because participants are almost always recruited from different sources—transgender participants are recruited from gender clinics; cisgender participants are recruited from universities, military, or the local community [29–31, 33–36, 44; but see 32]. Moreover, greater intelligence is associated with greater performance on spatial tasks [45, 46] and with greater hemisphere asymmetry [47]. Thus, measuring and controlling for intelligence is crucial for the comparison of task-domain and hemisphere-asymmetry effects in cis- and transgender individuals.

The present study assessed the extent to which task-domain and hemisphere-asymmetry effects in transgender individuals are moderated by assigned sex or gender identity, while controlling for participants’ intelligence, and maintaining homogeneity within and across participant groups with respect to sample source and age. All participants were recruited from the same undergraduate college population and were between 18–24 years old. Intelligence was measured using the Wechsler Abbreviated Scale of Intelligence (WASI) [48], which yields separate intelligence quotients (IQs) for spatial visualization and non-verbal reasoning (Performance IQ) and vocabulary knowledge and verbal reasoning (Verbal IQ), as well a composite score (Full IQ). Participants were categorized as cisfemale, cismale, or transmale based on their responses to questions about the sex they were assigned at birth and their gender identity. Transmale participants were assigned female at birth and did not identify as female.

Task-domain and hemisphere-asymmetry effects were examined with spatial and verbal activities presented to different hemispheres using a divided-visual field paradigm. The spatial activity was a two-dimensional mental-rotation task in which participants decided whether a rotated picture of an animal, presented briefly in the left or right visual field, was facing in the same or opposite direction as a previously-presented nonrotated version. Similar divided-visual field two-dimensional mental-rotation tasks have produced both task-domain and hemisphere-asymmetry effects in cisgender individuals [16–18]; thus, this task was appropriate for the current investigation. The verbal activity was a lexical-decision task in which participants decided whether a string of letters, presented briefly in the left or right visual field, formed a real word or not. Divided-visual field lexical decision is a well-validated measure of hemisphere asymmetries in verbal processing [49, 50], and although evidence for gender differences using this task is mixed, with some observing differences [20, 21] and others not [26, 28], the task was selected for the current investigation because it could be implemented similarly to the mental-rotation task, i.e., both tasks required participants to respond with 1 of 2 options to a visual item presented briefly in the periphery.

It was hypothesized that cismales would perform better on the mental-rotation task than cisfemales, and cisfemales would perform better on the lexical-decision task than cismales. In addition, it was hypothesized that cismales would exhibit greater differences between right and left hemisphere performance than cisfemales on both tasks, with greater right than left in the mental-rotation task and greater left than right in the lexical-decision task. No hypotheses were formed for transmale participants: they could exhibit task-domain and/or hemisphere-asymmetry effects that align with their assigned sex (female) or with their gender identity (male).

## Method

### Participants

Participants were recruited from Macalester College, a small liberal-arts college in Saint Paul, Minnesota, United States of America. A large number of transgender participants was recruited by advertising the study with materials specifically aimed at transgender individuals posted in spaces that attract transgender individuals. Non-native English speakers were excluded at recruitment in order to minimize variation during the verbal task. In addition, participants with known chromosomal or hormonal abnormalities, or who were undergoing any type of hormone therapy (not including birth control) were excluded. All participants were between 18–24 years of age and attending college currently or recently graduated (within a year). Participants gave their written consent to participate in the study in accordance with the guidelines established by the Code of Ethics of the World Medical Association (Declaration of Helsinki) and the Psychology Department Review Board at Macalester College. They were compensated with cash or extra credit in a course.

Participants were grouped based on answers they provided to two sets of questions. In the first set of questions, participants indicated what sex they were assigned at birth and responded “yes” or “no” to the following prompt: “Gender identity refers to how you feel inside and does not necessarily correspond to the sex you were assigned at birth or to how you look, act, or are perceived by others. Is your gender identity the same as the sex you were assigned at birth?”. This question revealed 55 participants who were assigned female at birth and whose gender identity was the same as that sex (cisfemale participants), 51 participants who were assigned male at birth and whose gender identity was the same as that sex (cismale participants), and 39 participants who were assigned female at birth and whose gender identity was not the same as that sex (transmale participants). Twelve participants responded that they were assigned male at birth and their gender identity is not the same as that sex, but these participants were excluded from the present study because there were so few of them compared to the other groups.

In the second question, participants were asked to select labels that they use to describe their gender identity from a list of 23 labels, including the option to write-in a label. Labels were feminine (e.g., “female”, “transwoman”, “male-to-female”), masculine (e.g., “male”, “transman”, “female-to-male”), or neutral (e.g., “non-binary”, “gender-nonconforming”, “androgynous”), and were used to decrease the heterogeneity of gender identity within each group. That is, cisfemale participants needed to select feminine label(s) and no masculine label(s); cismale participants needed to select masculine label(s) and no feminine label(s); and transmale participants needed to select masculine or neutral label(s) and no feminine label(s). These criteria lead to the exclusion of 1 cisfemale participant who did not include any feminine labels, 3 cismale participants (1 who did not include masculine labels and 2 who included feminine labels), and 7 transmale participants who included feminine labels.

Thus, the final participant groups consisted of 134 participants: 54 cisfemale participants (3 Asian/Pacific Islander, 1 Black, 4 Latinx/Hispanic, 7 mixed, 1 other, 1 US Indigenous, 37 White), 48 cismale participants (4 Asian/Pacific Islander, 2 Black, 2 Latinx/Hispanic, 3 mixed, 37 White), and 32 transmale participants (3 Black, 1 Latinx/Hispanic, 3 mixed, 1 other, 24 White). These 3 groups did not differ from each other in terms of age, *F*(2, 131) < 1, η_p_^2^ = .008, 95% CI [0, .052], or handedness, as assessed through the 10-item Edinburgh Inventory using laterality quotients, as described by Oldfield (1971) [51], *F*(2, 131) < 1, η_p_^2^ = .009, 95% CI [0, .054], or using number of left checks on the inventory, which provides slightly different information, *F*(2, 131) < 1, η_p_^2^ = .005, 95% CI [0, .042]. Descriptive statistics are provided in Table 1.

**Table 1:** Mean age and handedness for participant groups.

Group	Age	Laterality quotient	Left checks
Cisfemale	19.93 (1.06)	72.81 (41.47)	2.59 (4.10)
Cismale	19.83 (1.39)	67.24 (44.78)	2.92 (3.76)
Transmale	19.65 (1.10)	62.66 (40.99)	3.31 (3.53)

Age is provided in years. Laterality quotient and left checks are measures of handedness derived from the Edinburgh Inventory (Oldfield, 1971) [51]. Parentheses indicate standard deviation of the mean.

### Design

A 3 x 2 mixed-factorial design, in which participant group (cisfemale vs. cismale vs. transmale) was a between-subjects independent variable and hemisphere (left vs. right) was a within-subjects independent variable, was used for the mental-rotation and lexical-decision tasks. A sensitivity analysis using G*Power 3.1 [52] revealed that 134 participants is sufficient to detect a medium effect size of η_p_^2^ = .068, with an alpha of .05 and 80% power, in this design.

### Materials

Stimuli for the mental-rotation and lexical-decision tasks were presented on a MacBook Pro using PsyScope software [53]. Participants sat with their chin in a chinrest throughout each task in order to keep their eyes approximately 45 cm from the computer screen.

Stimuli for the mental-rotation task were 16 colored pictures of animals taken from Rossion and Pourtois (2004) [54] and selected such that half of the animals were clearly facing to the left and half were clearly facing to the right. Eight additional versions of each picture were created, first, by rotating the original in the clockwise direction 20˚, 40˚, 320˚, and 340˚, and then, by mirror-reversing each rotation so that the animal faced the opposite direction. Thus, 9 versions of each animal picture were used: the nonrotated original, 4 rotations facing the same direction, and 4 rotations facing the opposite direction. Pictures were presented on a white background and subtended 5.0˚ of widest (horizontal or vertical) visual angle. When presented in the left or right visual field, the middle of each picture was 6.7˚ from central fixation and the inner edge was at least 4.4˚ from central fixation.

Stimuli for the lexical-decision task were 64 emotionally-neutral words between 3–5 letters long taken from Bradley and Lang (1999) [55], and 64 pronounceable non-words created by rearranging the letters of each word (e.g., lamp → malp). Words and non-words were presented in black 36-point Helvetica font on a white background and subtended 2.4˚ x 0.8˚ of visual angle. When presented in the left or right visual field, the middle of each letter string was 5.9˚ from central fixation and the inner edge was at least 4.6˚ from central fixation.

Each task consisted of 128 trials, with half presented in the left visual field (to the right hemisphere) and half presented in the right (to the left hemisphere). Trials in the mental-rotation task began with a fixation cross presented in the center of the screen for 1000 ms. Participants were instructed to keep their eyes focused on the fixation cross whenever it was on the screen. The fixation cross was followed by a nonrotated picture of an animal presented in the center of the screen for 1500 ms. The central fixation cross then reappeared for 500 ms, and was followed by a rotated version of the same animal presented for 183 ms in the left or right visual field. This was followed by the central fixation cross, which remained on the screen until participants pushed a key responding whether the rotated animal was facing the same or opposite direction as the previously-presented nonrotated animal. Half of the trials presented in each visual field were facing the same direction and half were facing the opposite direction.

Trials in the lexical-decision task were structured similarly but did not include the central stimulus. Lexical-decision trials began with a fixation cross presented in the center of the screen for 1000 ms that participants were instructed to keep their eyes focused on. This fixation cross was followed by a letter string presented for 183 ms in the left or right visual field. The letter string was followed by the central fixation cross, which remained on the screen until participants pushed a key responding whether it formed a real word or not. Half of the trials presented in each visual field were words and half were nonwords.

Participants used the ‘o’ and ‘p’ keys on the computer keyboard to respond, and rested the index and middle fingers of their right hand on these keys throughout both tasks. The meaning of the keys was counterbalanced across participants such that half used ‘o’ for “same direction” in the mental-rotation task and “word” in the lexical-decision task, and half used ‘o’ for “opposite direction” in the mental-rotation task and “nonword” in the lexical-decision task. Trials in both tasks were presented in orders that were random with the constraint that no more than 3 of the same type of trial (left/right visual field or correct response type [e.g., same/opposite direction]) appeared consecutively. Participants completed 6 practice trials before each task to familiarize them with the brief lateralized presentations and response keys.

Percent correct and response times for correct responses were analyzed separately for each task. Participants who did not achieve 60% percent correct or higher on either task were excluded from the analysis of that task. Response times that were under 250 ms or above 2.5 standard deviations of the participant’s mean were excluded from analyses of response times.

Intelligence was assessed using the WASI, administered and scored identically to the manner described by Wechsler (1999) [48] in order to yield Performance IQ, Verbal IQ, and Full IQ scores. The WASI consists of 4 subtests, Vocabulary, Block Design, Similarities, and Matrix Reasoning, completed in that order. Performance IQ is based on the Block Design and Matrix Reasoning subtests; Verbal IQ is based on the Vocabulary and Similarities subtests; Full IQ is based on all 4 subtests. The entire test takes about 30 minutes.

### Procedure

Participants were tested individually in a private room with the experimenter. After completing a consent form describing the nature of the study, participants engaged in the mental-rotation and lexical-decision tasks, presented in counterbalanced order. After completing the mental-rotation and lexical-decision tasks, participants answered questions about their assigned sex and gender identity, as well as about aspects of their medical history and demographic, including handedness (see Participants section). Participants then completed the WASI. The entire study took approximately 1 hour.

## Results

### Intelligence

**Fig 1:**
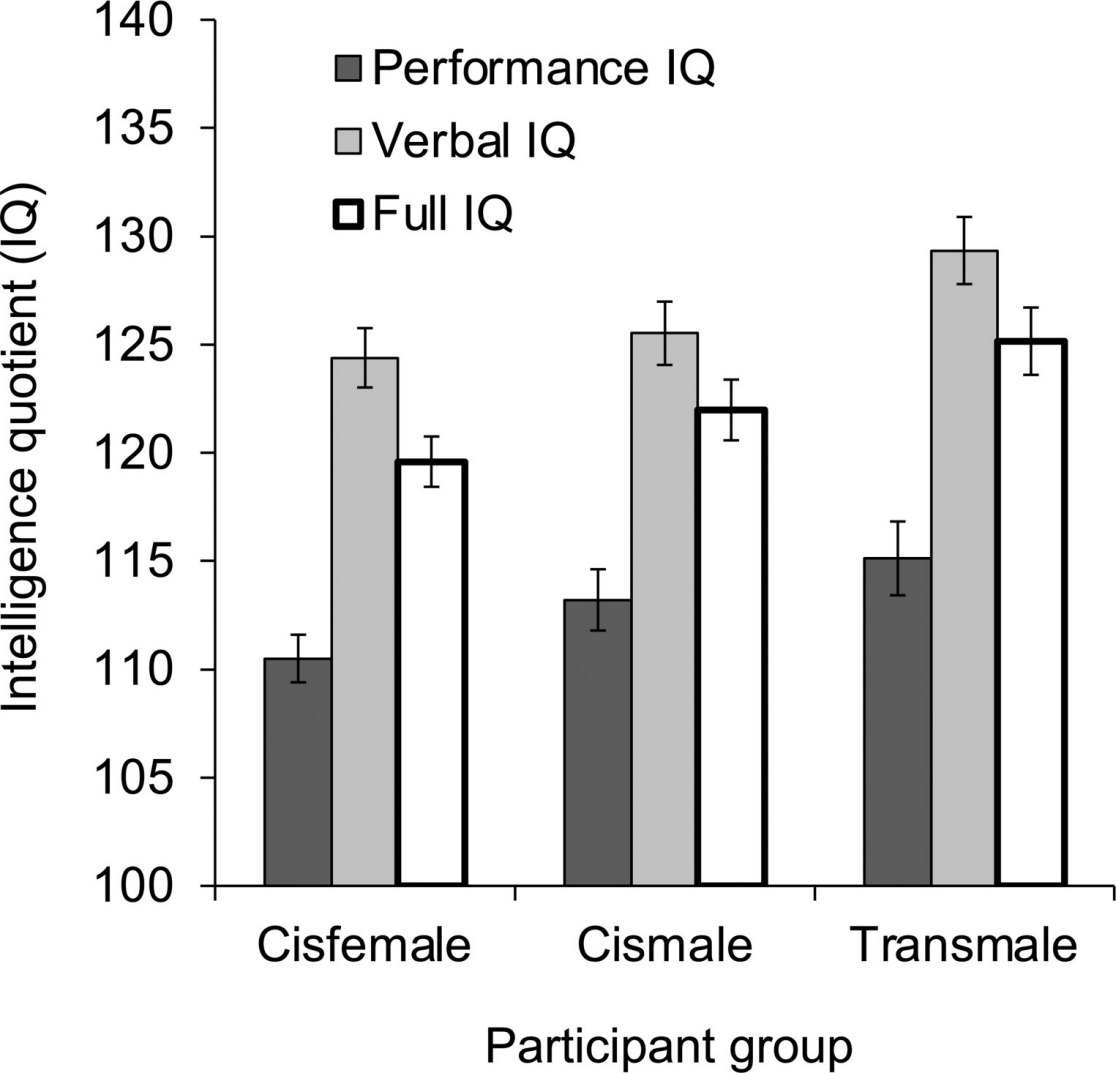
Intelligence quotient. Intelligence quotient (IQ) displayed as a function of participant group (cisfemale, cismale, transmale) and type of intelligence (Performance IQ, Verbal IQ, Full IQ). Error bars indicate standard error of the mean.

IQ scores were analyzed in a 3 x 2 repeated-measures analysis of variance (ANOVA) with participant group (cisfemale vs. cismale vs. transmale) as a between-subjects independent variable and type of intelligence (Performance IQ vs. Verbal IQ) as a within-subjects independent variable (see Fig 1). Most important, the main effect of participant group was significant, *F*(2, 131) = 3.62, *p* = .029, η_p_^2^ = .052, 95% CI [0, .133]. Post-hoc *t* tests revealed higher scores in transmale participants (*M* = 122, *SD* = 8) than cisfemale participants (*M* = 117, *SD* = 7), *t*(84) = 2.84, *p* = .006, *d* = .656, 95% CI [.226, 1.12], but no difference between transmale participants and cismale participants (*M* = 119, *SD* = 9), *t*(78) = 1.52, *p* = .134, *d* = .342, 95% CI [-.104, .798], or between cisfemale participants and cismale participants, *t*(100) = 1.20, *p* = .233, *d* = .250, 95% CI [-.141, .640]. The main effect of type of intelligence was also significant, *F*(1, 131) = 224.37, *p* < .001, η_p_^2^ = .631, 95% CI [.532, .700], with higher Verbal IQ scores (*M* = 126, *SD* = 10) than Performance IQ scores (*M* = 113, *SD* = 9). Critically, however, this difference did not vary by participant group, as evidenced by the lack of participant group x type of intelligence interaction, *F*(2, 131) < 1, η_p_^2^ = .007, 95% CI [0, .047].

Since Full IQ scores are slightly different from the average of Performance and Verbal IQ scores, Full IQ scores were analyzed separately in a one-way ANOVA assessing participant group (cisfemale vs. cismale vs. transmale; see Fig 1). In line with the analysis of Performance and Verbal IQ above, the effect of participant group was significant, *F*(2, 131) = 3.83, *p* = .024, η_p_^2^ = .055, 95% CI [0, .137], with post-hoc *t* tests revealing higher scores in transmale participants (*M* = 125, *SD* = 9) than cisfemale participants (*M* = 120, *SD* = 9), *t*(84) = 2.89, *p* = .005, *d* = .539, 95% CI [.109, .999], but no difference between transmale and cismale participants (*M* = 122, *SD* = 10), *t*(78) = 1.49, *p* = .141, *d* = .307, 95% CI [-.139, .761], or between cisfemale and cismale participants, *t*(100) = 1.32, *p* = .190, *d* = .211, 95% CI [-.180, .600].

### Mental rotation

**Fig 2:**
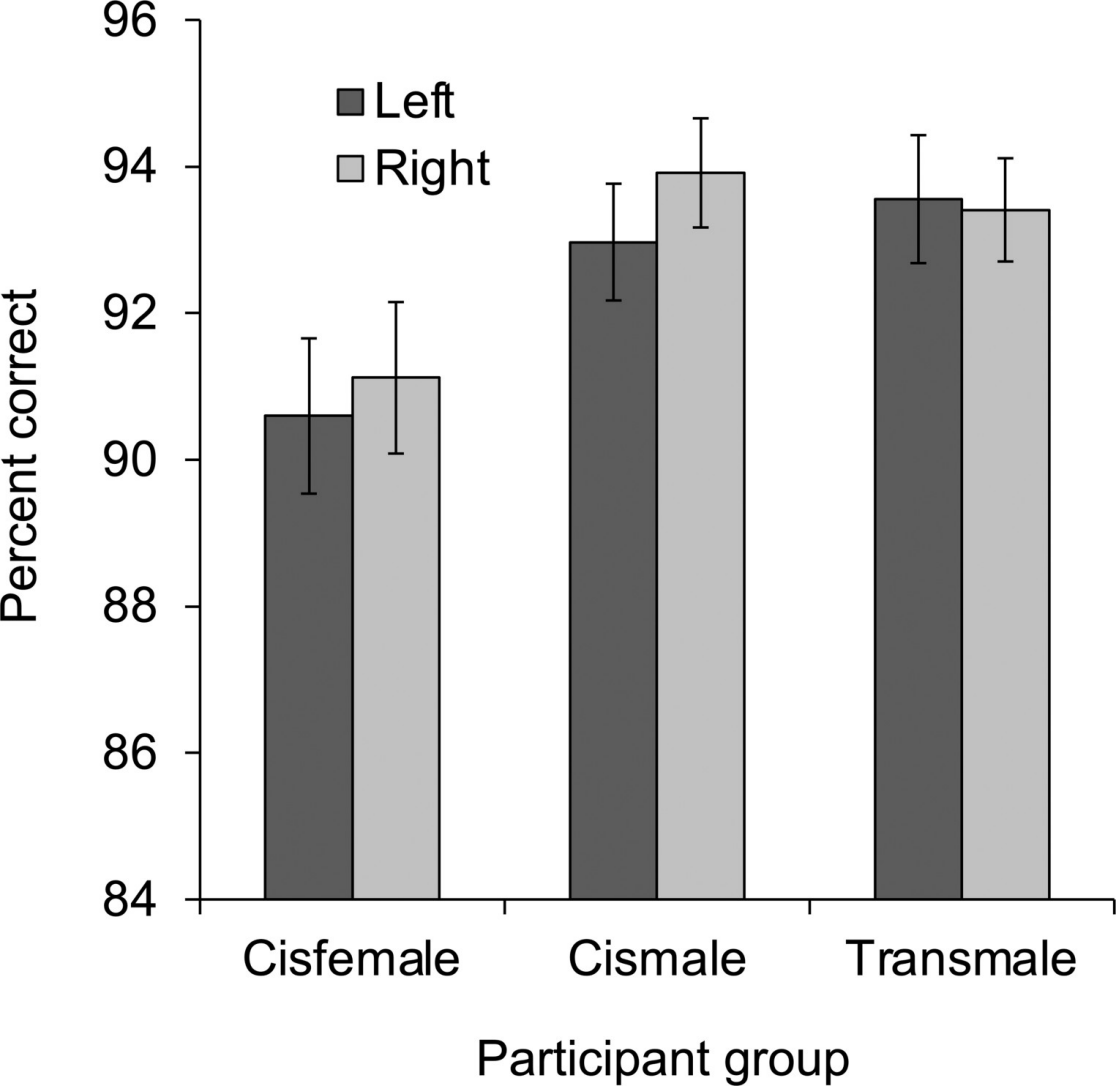
Mental-rotation performance. Percent correct in the mental-rotation task displayed as a function of participant group (cisfemale, cismale, transmale) and hemisphere (left, right). Error bars indicate standard error of the mean.

Percent correct in the mental-rotation task was analyzed in a 3 x 2 repeated-measures ANOVA with participant group (cisfemale vs. cismale vs. transmale) as a between-subjects independent variable and hemisphere (left vs. right) as a within-subjects independent variable (see Fig 2). Most important, this analysis revealed a main effect of participant group, *F*(2, 131) = 3.17, *p* = .045, η_p_^2^ = .046, 95% CI [0, .126]. Post-hoc *t* tests revealed that cismale participants (*M* = 93%, *SD* = 5%) performed better than cisfemale participants (*M* = 91%, *SD* = 7%), *t*(100) = 2.08, *p* = .040, *d* = .326, 95% CI [-.067, .716]. Transmale participants (*M* = 93%, *SD* = 4%) also performed marginally better than cisfemale participants, *t*(84) = 1.88, *p* = .063, *d* = .320, 95% CI [-.111, .769], and did not differ from cismale participants, *t*(78) < 1, *d* = .000, 95% CI [-.447, .447]. Neither the main effect of hemisphere, *F*(1, 131) = 1.07, *p* = .303, η_p_^2^ = .008, 95% CI [0, .063], nor the interaction of participant group x hemisphere, *F*(2, 131) < 1, η_p_^2^ = .009, 95% CI [0, .054], were significant.

In order to assess the extent to which the effect of participant group was influenced by intelligence, as well as directly compare the effects of gender identity and assigned sex on the group difference, a 3-step, stepwise multiple-regression analysis was conducted using percent correct averaged across hemisphere as the dependent variable, and (1) gender identity (female = 1; male = -1), (2) assigned sex (female = 1; male = -1), and (3) Full IQ as the independent variables. In line with results from the ANOVA, gender identity accounted for a significant proportion of the variance in percent correct when entered in step 1, *R*^2^ = .050, 95% CI [.003, .143], *F*(1, 132) = 6.91, *p* = .010, and this did not change when assigned sex was entered in step 2, Δ*R*^2^ = .000, *F*(1, 131) < 1. When Full IQ was added in step 3, however, the change in *R*^2^ was significant, Δ*R*^2^ = .150, *F*(1, 130) = 24.32, *p* < .001. Individual standardized coefficients from this step reveal a positive effect of Full IQ, *b* = .003 (± .001), 95% CI [.002, .004], ß = .398, *t*(130) = 4.93, *p* < .001, but no effect of gender identity, *b* = -.006 (± .006), 95% CI [-.018, .006], ß = -.106, *t*(130) = 1.04, *p* = .300, or assigned sex, *b* = -.004 (± .006), 95% CI [-.016, .008], ß = -.063, *t*(130) < 1. Step 3 was also conducted using Performance IQ instead of Full IQ, due to its more specific association with visual-spatial processing. Results were highly similar: the change in *R*^2^ due to Performance IQ was significant, Δ*R*^2^ = .189, *F*(1, 130) = 32.34, *p* < .001, and there was a positive effect of Performance IQ, *b* = .003 (± .000), 95% CI [.002, .004], ß = .444, *t*(130) = 5.69, *p* < .001, with no effects of gender identity, *b* = -.007 (± .006), 95% CI [-.019, .005], ß = -.115, *t*(130) = 1.16, *p* = .246, or assigned sex, *b* = -.003 (± .006), 95% CI [-.014, .009], ß = -.042, *t*(130) < 1. Complete results from the regression analyses are presented in Table 2.

**Table 2:** Stepwise multiple-regression analysis of percent correct averaged across hemisphere in mental-rotation task.

Step	Variable	*R* ^2^	*F*	ß	*t*
1	Gender	.050	6.91*	-.223	2.63*
2	Gender	.050	3.43*	-.225	2.08*
	Assigned sex			.003	.024
3	Gender	.200	10.80**	-.106	1.04
	Assigned sex			-.063	.632
	Full IQ			.398	4.93**
3	Gender	.239	13.61**	-.115	1.16
	Assigned sex			-.042	.430
	Performance IQ			.444	5.69**

* = *p* < .05

** = *p* < .001

Response times in the mental-rotation task were analyzed in the same way as percent correct, after excluding incorrect responses and outliers below 250 ms or above 2.5 standard deviations of the participant’s mean. No significant effects were observed in the 3 x 2 repeated-measures ANOVA with participant group (cisfemale vs. cismale vs. transmale) and hemisphere (left vs. right), all *p*s > .169, or in the 3-step, stepwise multiple-regression analyses of response times averaged across hemisphere, all *p*s > .096. Complete results from the analyses of response times in the mental-rotation task are provided as supporting information (see S1 Table).

### Lexical decision

**Fig 3:**
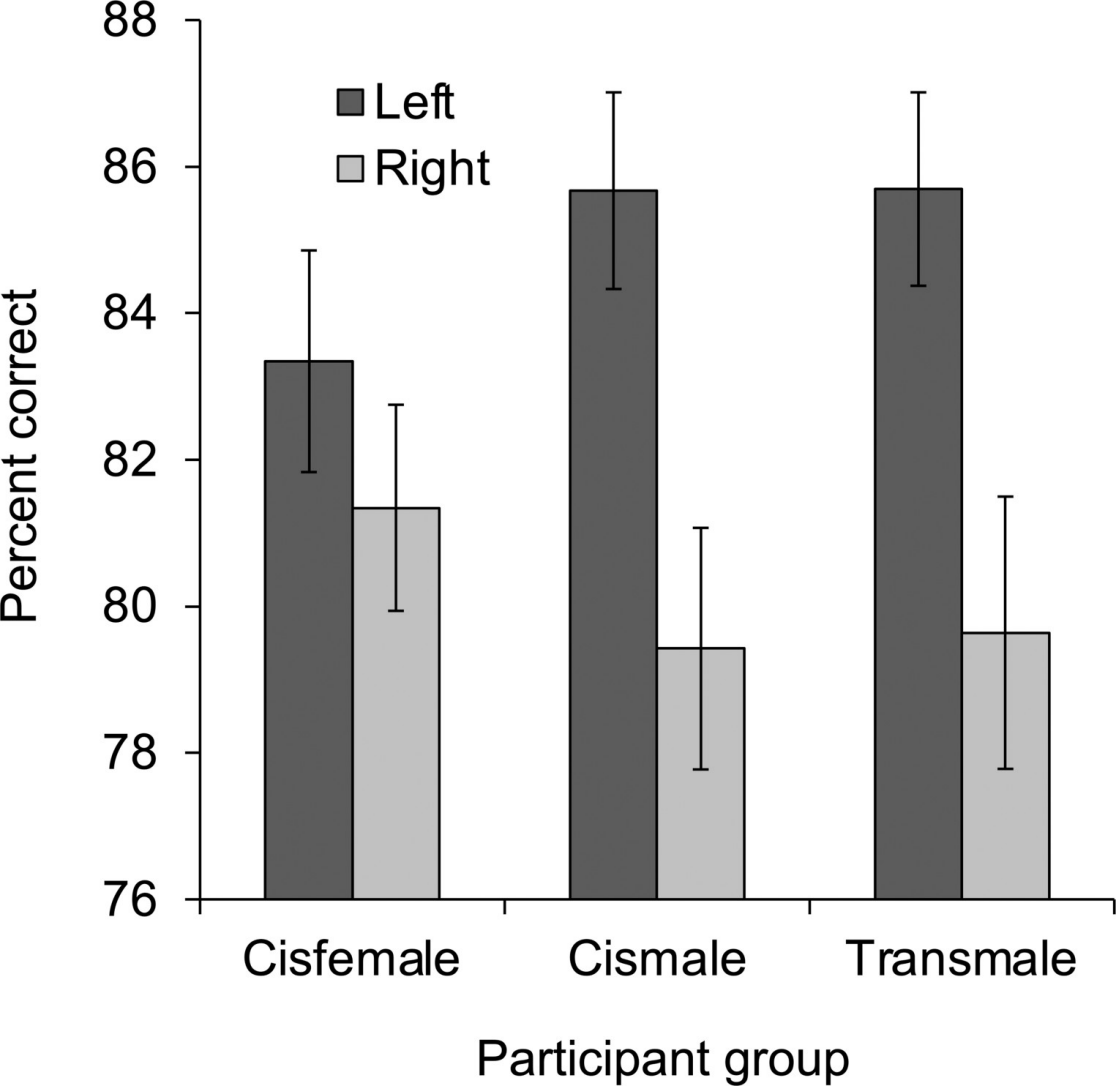
Lexical-decision performance. Percent correct in the lexical-decision task displayed as a function of participant group (cisfemale, cismale, transmale) and hemisphere (left, right). Error bars indicate standard error of the mean.

Five participants (4 cisfemale; 1 cismale) did not achieve 60% or higher accuracy on the lexical-decision task and were excluded from the analysis of this task. As in the analysis of mental-rotation performance, percent correct during lexical decision was analyzed in a 3 x 2 repeated-measures ANOVA with participant group (cisfemale vs. cismale vs. transmale) as a between-subjects independent variable and hemisphere (left vs. right) as a within-subjects independent variable (see Fig 3). Overall, percent correct was higher for trials presented to the left hemisphere (*M* = 85%, *SD* = 9%) than for trials presented to the right hemisphere (*M* = 80%, *SD* = 11%), *F*(1, 126) = 46.46, *p* < .001, η_p_^2^ = .269, 95% CI [.146, .384], for the main effect of hemisphere. Most important, this main effect was qualified by a significant interaction of participant group x hemisphere, *F*(2, 126) = 4.46, *p* = .013, η_p_^2^ = .066, 95% CI [.003, .154]. Post-hoc analyses revealed a greater difference between the left and right hemispheres in cismale participants (*M*s = 86% [*SD* = 9%] vs. 79% [*SD* = 11%]) than cisfemale participants (*M*s = 83% [*SD* = 11%] vs. 81% [*SD* = 10%]), *F*(1, 95) = 7.28, *p* = .008, η_p_^2^ = .071, 95% CI [.005, .185], for the 2 x 2 interaction of participant group (cisfemale vs. cismale) by hemisphere. Transmale participants also exhibited a greater difference between the left and right hemispheres (*M*s = 86% [*SD* = 7%] vs. 80% [*SD* = 11%]) than cisfemales, *F*(1, 80) = 5.42, *p* = .022, η_p_^2^ = .063, 95% CI [0, .185], for the 2 x 2 interaction of participant group (cisfemale vs. transmale) by hemisphere. Moreover, transmale participants did not differ from cismale participants, *F*(1, 77) < 1, η_p_^2^ = .000, 95% CI [0, .039], for the 2 x 2 interaction of participant group (cismale vs. transmale) by hemisphere. The main effect of participant group from the 3 x 2 repeated-measures ANOVA was not significant, *F*(2, 126) < 1, η_p_^2^ = .000, 95% CI [0, 0].

In order to assess the extent to which the interaction of participant group x hemisphere was influenced by intelligence, as well as directly compare the effects of gender identity and assigned sex on the magnitude of the hemisphere asymmetry, a 3-step, stepwise multiple-regression analysis was conducted using the difference between left- and right-hemisphere percent correct (left–right) as the dependent variable, and (1) gender identity (female = 1; male = -1), (2) assigned sex (female = 1; male = -1), and (3) Full IQ as the independent variables. In line with results from the ANOVA, gender identity accounted for a significant proportion of the variance in percent correct when entered in step 1, *R*^2^ = .066, 95% CI [.008, .168], *F*(1, 127) = 8.94, *p* = .003, and this did not change when assigned sex was entered in step 2, Δ*R*^2^ = .000, *F*(1, 126) < 1. Moreover, the addition of Full IQ in step 3 did not affect this pattern, Δ*R*^2^ = .000, *F*(1, 125) < 1, with individual standardized coefficients from this step revealing a negative effect of gender identity, *b* = -.020 (± .009), 95% CI [-.038, -.003], ß = -.251, *t*(125) = 2.26, *p* = .026, and no effects of assigned sex, *b* = -.001 (± .009), 95% CI [-.019, .017], ß = -.014, *t*(125) < 1, or Full IQ, *b* = .000 (± .001), 95% CI [-.002, .001], ß = -.014, *t*(125) < 1. Step 3 was also conducted using Verbal IQ instead of Full IQ, due to its more specific association with verbal processing. Results were highly similar: there was no significant change in *R*^2^ due to Verbal IQ, Δ*R*^2^ = .005, *F*(1, 125) < 1, and there was a significant effect of gender identity, *b* = -.021 (± .009), 95% CI [-.039, -.004], ß = -.263, *t*(125) = 2.39, *p* = .018, with no effects of assigned sex, *b* = .000 (± .009), 95% CI [-.018, .017], ß = -.003, *t*(125) < 1, or Verbal IQ, *b* = -.001 (± .001), 95% CI [-.002, .001], ß = -.071, *t*(125) < 1. Complete results from the regression analyses are presented in Table 3.

**Table 3:** Stepwise multiple-regression analysis of difference between left and right-hemisphere percent correct in lexical-decision task.

Step	Variable	*R* ^2^	*F*	ß	*t*
1	Gender	.066	8.94*	-.256	2.99*
2	Gender	.066	4.45*	-.247	2.29*
	Assigned sex			-.016	.150
3	Gender	.066	2.95*	-.251	2.26*
	Assigned sex			-.014	.127
	Full IQ			-.014	.162
3	Gender	.071	3.18*	-.263	2.39*
	Assigned sex			-.003	.032
	Verbal IQ			-.071	.811

* = *p* < .05

Response times in the lexical-decision task were analyzed in the same way as percent correct, after excluding incorrect responses and outliers below 250 ms or above 2.5 standard deviations of the participant’s mean. The 3 x 2 repeated-measures ANOVA with participant group (cisfemale vs. cismale vs. transmale) as a between-subjects independent variable and hemisphere (left vs. right) as a within-subjects independent variable revealed a main effect of hemisphere in which response times were faster for trials presented to the left hemisphere (*M* = 820 ms, *SD* = 151 ms) than for trials presented to the right hemisphere (*M* = 855 ms, *SD* = 176 ms), *F*(1, 126) = 38.88, *p* < .001, η_p_^2^ = .236, 95% CI [.117, .352]. Neither the main effect of participant group, *F*(2, 126) < 1, η_p_^2^ = .008, 95% CI [0, .052], nor the participant group x hemisphere interaction, *F*(2, 126) < 1, η_p_^2^ = .002, 95% CI [0, .021], were significant. The 3-step, stepwise multiple-regression analyses of the difference between left- and right-hemisphere response times (right–left) did not produce any significant effects, all *p*s > .597. Complete results from the analyses of response times in the lexical-decision task are provided as supporting information (see S1 Table).

## Discussion

The present research examined the extent to which transgender individuals’ functional brain organization resembles that of their assigned sex or gender identity by comparing cisfemale, cismale, and transmale participants in terms of task-domain and hemisphere asymmetry effects, while controlling for intelligence. Results do not support task-domain effects when intelligence is accounted for; however, a hemisphere-asymmetry effect was observed in the verbal domain that aligns with gender identity and not assigned sex. Findings and their implications are discussed below.

The mental-rotation task revealed results that at first seem to support a task-domain effect that aligns with gender identity and not assigned sex. Cismale and transmale participants performed better than cisfemale participants and did not differ from each other. This finding contrasts with results from previous studies that show effects of assigned sex on spatial processing [29–31] and is more in line with work suggesting gender identity is important [34]. Critically, however, the effect of gender identity in the present study disappeared when intelligence was accounted for. Intelligence was positively correlated with mental-rotation performance, and transmale participants had higher intelligence scores than cisfemales, who were similar to cismales. Thus, improved mental-rotation performance in transmale individuals was attributable to their higher intelligence rather than their gender identity or assigned sex.

A positive relationship between intelligence and mental-rotation performance has been observed previously in cisgender individuals [45, 46]. The present results extend this finding to transmale individuals and suggest that previous findings of greater mental-rotation performance in cisgender males than females [16–18] may be due, at least in part, to differences in intelligence. As noted by Hines (2020) [4], and in the Introduction, differences in intelligence between gender-identity or assigned-sex groups may emerge due to selection biases that favor females and high intelligence. As such, the present results underscore the importance of measuring and controlling for intelligence in studies comparing these groups.

Interestingly, the positive effect of intelligence on mental-rotation performance in the present study was similar regardless of whether Full IQ or Performance IQ was used as the measure of intelligence. The WASI measure of Performance IQ assesses visual-spatial abilities that are directly relevant for mental rotation, while the Verbal IQ measure, which is included in Full IQ, assesses vocabulary knowledge and verbal reasoning skills that seem less relevant. As such, one might expect Performance IQ to be a better predictor of mental rotation than Full IQ. That Full IQ also predicts mental-rotation performance is not a novel finding however—Anomal et al. (2020) [45] observed better mental-rotation performance in participants with higher compared to lower intelligence scores that included a verbal component much like the present Full IQ scores. Moreover, increased ERP amplitude over the right compared to left frontal lobe during mental rotation was predicted by intelligence, working memory, and verbal comprehension, suggesting a similar (frontal) locus for these processes. From this perspective, it is not surprising that Full IQ predicted mental-rotation performance as well as Performance IQ in the present study.

In contrast to predictions, results from the mental-rotation task did not reveal a hemisphere-asymmetry effect. Previous studies using similar divided-visual field mental-rotation tasks have observed a right-hemisphere advantage that is greater in cisgender males than females [16–18], however none of the participant groups exhibited a difference between right- and left-hemisphere presentations in the present study. A possible explanation for this discrepancy is the complexity of the stimuli used. Research suggests that mental-rotation of simple stimuli relies on a holistic strategy while rotation of more complex stimuli relies on a parts-based strategy [56, 57], and moreover, that holistic and parts-based strategies are linked to right and left-hemisphere systems, respectively [58, 59]. Unlike previous studies that used relatively simple line drawings of stick figures [17] or abstract shapes [16, 18], the present study used colored pictures of animals that varied in their complexity. Thus, it is possible that different pictures recruited right and left-hemisphere systems variably depending on their complexity, resulting in no difference between hemispheres overall. Of course, this explanation is speculative and requires direct testing to confirm or deny.

The lexical-decision task, in contrast, did reveal a hemisphere-asymmetry effect, and this effect aligned with gender identity and not assigned sex. Cismale and transmale participants exhibited a greater left- over right-hemisphere advantage than cisfemale participants. Critically, accounting for intelligence scores did not change this effect. Thus, the greater left-hemisphere advantage in cismale and transmale participants than cisfemales is associated with gender identity rather than assigned sex or intelligence. That gender identity is important is in line with results from Govier et al. (2010) [35] in which the left-hemisphere advantage during a dichotic listening task depended on gender identity. The present study extends this finding to another verbal task and rules out the possibility that group difference in intelligence are influential. Moreover, similar to the mental-rotation task, findings did not change depending on what type of intelligence was examined (Full IQ vs. Verbal IQ)—neither type changed the effect of gender identity on hemisphere asymmetry.

Of note, no effect of task-domain was observed in the lexical-decision task. Cisfemales did not perform better than cismales, and there was no difference between groups when hemisphere was ignored. Previous research suggests that cisfemales perform better on verbal tasks than cismales, however this research is in terms of verbal memory [11], verbal fluency [12], and reading achievement [13], rather than lexical decision, and direct tests of task-domain effects in lexical-decision tasks have not revealed gender differences [26, 28]. As described in the Introduction, lexical decision was used in the present study because it could be implemented in divided-visual field similarly to the mental-rotation task, and because it is a well-validated measure of hemisphere asymmetries in verbal processing [49, 50]. Critically, however, future research using another measure of verbal processing, such as verbal fluency, memory, or reading, may be more informative regarding task-domain effects.

The lack of task-domain effect in either the verbal or spatial task in the present study highlights the value of examining potential hemisphere asymmetries when comparing different gender or assigned-sex groups. Indeed, hemisphere-asymmetry effects may be more sensitive than task-domain effects to differences between groups based on gender identity. However, hemisphere-asymmetry effects may not be the most robust tool for evaluating functional neural organization, as in the present study the effect was only observed during the lexical-decision task—not during the mental-rotation task—and only in the percent-correct measure—not in the response times. Response times may not have been as sensitive to differences as percent correct because task instructions emphasized speed over accuracy, and fewer trials were included in the response-time analysis (due to the exclusion of incorrect trials) reducing its reliability. Most important though, response times did not contradict the percent-correct data, belying any tradeoff between accuracy and speed. Nonetheless, direct assessments of hemisphere asymmetries using neuroimaging or neurophysiological measures are generally more sensitive and robust than indirect behavioral measures, and future research would benefit from the use of these methods.

As described in the Introduction, previous research examining task-domain and hemisphere-asymmetry effects in transgender individuals has been limited by small sample sizes, large age ranges, and age and education confounds, as well as by sampling biases that could lead to confounding effects of intelligence. The present study overcame these limitations by recruiting adequate samples of cis- and transgender participants from the same relatively homogeneous college population, and by measuring and controlling for intelligence. In addition, the present study increased within-group homogeneity by asking about the labels that participants use to describe their gender identity and excluding people who did not choose labels that were clearly female, for cisfemale participants, clearly male, for cismale participants, or clearly not female, for transmale participants. Previous studies have not used labels to verify assumptions about participants’ gender identities, and thus, may have categorized/included participants incorrectly. This could be especially problematic in studies that use small numbers of participants per group, as several do [30, 31, 33, 34, 36]. As such, this represents another way in which the present research improves on previous.

Although the increased homogeneity within and across participant groups strived for in the present study had the benefit of controlling for a variety of potentially misleading influences, it also limited the study, most notably by prohibiting the inclusion of transgender participants assigned male at birth. As described in the Participants section, the present study only recruited 12 transgender participants who were assigned male at birth, compared to 39 who were assigned female, and therefore, did not include a transfemale group in the analyses. This difference in response rates is likely due to differences that exist in the specific college population recruited from rather than differences in the population at large. Indeed, other studies observe equal [60] or higher [61] rates of transgender people assigned male at birth compared to female. As such, sampling from other populations would likely reveal more transfemale participants than the present study did. Critically, it cannot be assumed that the moderation of hemisphere asymmetries by gender identity and not assigned sex observed for transmales in the present study would be observed for transfemales. Thus, it will be essential to include transfemale as well as transmale participants in future research in order to test the extent to which conclusions can be generalized to all transgender individuals.

Another potential limitation of the present study was the inclusion of participants taking hormonal birth control. Although participants undergoing other types of hormone therapy were excluded, participants taking hormonal contraception were included in order to increase the number of people in the population that were eligible for the study, and as such, 27 cisfemale and 9 transmale participants taking hormonal birth control participated. Hormonal contraception has been associated with structural and functional changes throughout the brain [62] as well as with performance changes in spatial and verbal processing [63–65], although the extent and direction of the changes is affected by type of contraceptive (androgenic or anti-androgenic) [66]. Thus, it is possible that the inclusion of these participants altered the patterns of results observed. To examine this possibility, data were reanalyzed excluding participants taking hormonal birth control (see S2 Table). Importantly, although not all effects were significant in these reduced-power analyses, qualitative patterns were similar to those observed in the full analysis, suggesting that hormonal contraception may not have played a role in the present study. However, future research may want to exclude hormonal-contraception users initially; or alternatively, directly compare those taking hormonal birth control to those not, while taking account of contraception type and menstrual-cycle phase.

In conclusion, the present study provides evidence for a hemisphere-asymmetry effect in the verbal domain that aligns with gender identity and not assigned sex, and supports the idea that transmale individuals’ functional neuroanatomy is organized in alignment with expressed gender identity. This research improved on previous research in a number of ways, most notably by controlling for intelligence. Although intelligence affected the task-domain effect in the spatial task, the hemisphere-asymmetry effect was not influenced by variation in intelligence. Importantly, the present results must not be misconstrued as having a diagnostic application (i.e., as a tool to evaluate gender identity). An individual’s stated gender identity is the premise from which research questions must begin, rather than a question that this type of research can address. Nonetheless, hemisphere asymmetries may be a useful tool for assessing functional neural organization in different gender-identity groups provided that the appropriate caution is exercised.

## Supporting information

S1 Table Results from analyses of response times. (PDF)

S2 Table Results from analyses excluding participants taking hormonal contraception. (PDF)
