# Supplementary material for: Task-domain and hemisphere-asymmetry effects in cisgender and transmale individuals
Source: PLoS One. 2021 Dec 7;16(12):e0260542. doi: 10.1371/journal.pone.0260542 (PMC8651105; doi:10.1371/journal.pone.0260542)
Supplement: S1 Table — (PDF) [file pone.0260542.s001.pdf]

S1 Tables. Results from Analyses of Response Times.

Mean Response Time in Mental-Rotation Task.

Group	Hemisphere	
	Left	Right
Cisfemale	962 (215)	961 (218)
Cismale	880 (189)	880 (193)
Transmale	924 (254)	925 (262)

Response times are provided in milliseconds. Parentheses indicate standard deviation of the mean.

Stepwise Multiple-Regression Analysis of Response Time Averaged Across Hemisphere in Mental-Rotation Task.

Step	Variable	R^2	F	ß	t
1	Gender	.021	2.79	.144	1.67
2	Gender	.027	1.80	.083	.765
	Assigned sex			.098	.901
3	Gender	.029	1.29	.098	.869
	Assigned sex			.090	.818
	Full IQ			.048	.545
3	Gender	.031	1.39	.067	.299
	Assigned sex			.105	.959
	Performance IQ			-.068	.766

Mean Response Time in Lexical-Decision Task.

	Hemisphere	
Group	Left	Right
Cisfemale	836 (180)	875 (212)
Cismale	808 (113)	844 (135)
Transmale	811 (154)	843 (170)

Response times are provided in milliseconds. Parentheses indicate standard deviation of the mean.

Stepwise Multiple-Regression Analysis of Difference Between Left and Right-Hemisphere Response Time in Lexical-Decision Task.

Step	Variable	R^2	F	ß	t
1	Gender	.001	.152	.035	.390
2	Gender	.002	.101	.050	.447
	Assigned sex			-.025	.227
3	Gender	.003	.130	.061	.529
	Assigned sex			-.032	.281
	Full IQ			.040	.436
3	Gender	.002	.068	.051	.447
	Assigned sex			-.026	.232
	Verbal IQ			.005	.054
