# Supplementary material for: Task-domain and hemisphere-asymmetry effects in cisgender and transmale individuals
Source: PLoS One. 2021 Dec 7;16(12):e0260542. doi: 10.1371/journal.pone.0260542 (PMC8651105; doi:10.1371/journal.pone.0260542)
Supplement: S2 Table — (PDF) [file pone.0260542.s002.pdf]

S2 Tables. Results from Analyses Excluding Participants Taking Hormonal Contraception.

Mean Intelligence Quotient (IQ).

Group	Performance IQ	Verbal IQ	Full IQ
Cisfemale	111 (9)	125 (10)	120 (10)
Cismale	113 (10)	126 (10)	122 (10)
Transmale	118 (7)	131 (9)	128 (7)

Parentheses indicate standard deviation of the mean. A two-way repeated-measures ANOVA revealed main effects of intelligence type (performance vs. verbal), $F(1, 95) = 173.99$, $p < .001$, $\eta_p^2 = .647$, 95% CI [.530, .722], and group, $F(2, 95) = 5.43$, $p = .006$, $\eta_p^2 = .103$, 95% CI [0, .215]. A one-way ANOVA assessing full IQ revealed an effect of group, $F(2, 95) = 5.67$, $p = .005$, $\eta_p^2 = .107$, 95% CI [.012, .220].

Mean Percent Correct and Response Time in Mental-Rotation Task.

Group	Percent correct		Response time (ms)	
	Left hemisphere	Right hemisphere	Left hemisphere	Right hemisphere
Cisfemale	90 (10)	91 (9)	966 (175)	964 (192)
Cismale	93 (6)	94 (5)	880 (189)	880 (193)
Transmale	95 (4)	94 (4)	961 (269)	965 (282)

Parentheses indicate standard deviation of the mean. Two-way repeated-measures ANOVAs for percent correct and response times revealed a main effect of group in percent correct, $F(2, 95) = 3.37$, $p = .038$, $\eta_p^2 = .066$, 95% CI [0, .167], and no other significant effects, all *p*s > .123.

Stepwise Multiple-Regression Analysis of Mental-Rotation Performance Averaged Across Hemisphere.

Step	Variable	Percent correct				Response time			
		R^2	F	ß	t	R^2	F	ß	t
1	Gender	.067	6.87*	-.258	2.62*	.015	1.49	.124	1.22
2	Gender	.071	3.61*	-.305	2.46*	.040	1.96	.005	.041
	Assigned sex			.077	.621			.196	1.55
3	Gender	.250	10.45**	-.130	1.10	.043	1.40	.028	.213
	Assigned sex			-.072	.622			.176	1.34
	Full IQ			.448	4.74**			.060	.559
3	Gender	.268	11.47**	-.129	1.11	.042	1.36	.022	.169
	Assigned sex			-.054	.473			.183	1.41
	Performance IQ			.466	5.03**			.046	.435

* = $p < .05$; ** = $p < .001$

Mean Percent Correct and Response Time in Lexical-Decision Task.

	Percent correct		Response time (ms)	
Group	Left hemisphere	Right hemisphere	Left hemisphere	Right hemisphere
Cisfemale	87 (9)	83 (11)	854 (194)	909 (244)
Cismale	86 (9)	79 (11)	808 (113)	844 (135)
Transmale	87 (7)	80 (10)	805 (118)	834 (132)

Parentheses indicate standard deviation of the mean. Two-way repeated-measures ANOVAs for percent correct and response times revealed main effects of hemisphere in both measures, percent correct: $F(1, 92) = 47.32$, $p < .001$, $\eta_p^2 = .340$, 95% CI [.188, .466]; response time: $F(1, 92) = 28.36$, $p < .001$, $\eta_p^2 = .236$, 95% CI [.099, .369]. No other effects reached significance, all ps > .266.

Stepwise Multiple-Regression Analysis of Difference Between Left and Right-Hemisphere Lexical-Decision Performance.

		Percent correct				Response time			
Step	Variable	R^2	F	ß	t	R^2	F	ß	t
1	Gender	.026	2.44	-.160	1.56	.018	1.73	.135	1.32
2	Gender	.026	1.24	-.179	1.40	.019	.912	.160	1.25
	Assigned sex			.032	.252			-.042	.328
3	Gender	.035	1.10	-.216	1.61	.021	.635	.173	1.28
	Assigned sex			.064	.483			-.053	.399
	Full IQ			-.099	.907			.035	.317
3	Gender	.040	1.28	-.216	1.64	.020	1.73	.164	1.24
	Assigned sex			.068	.518			-.046	.346
	Verbal IQ			-.123	1.16			.013	.124
